# A checklist of gymnosperm-feeding leafminers (Arthopoda, Insecta) in North America and Europe

**DOI:** 10.3897/BDJ.10.e91313

**Published:** 2022-09-28

**Authors:** Taibin Chen, Xiaohua Dai, Charles Eiseman

**Affiliations:** 1 Leafminer Group, School of Life Sciences, Gannan Normal University, Ganzhou, China Leafminer Group, School of Life Sciences, Gannan Normal University Ganzhou China; 2 National Navel-Orange Engineering Research Center, Ganzhou, China National Navel-Orange Engineering Research Center Ganzhou China; 3 Ganzhou Key Laboratory of Nanling Insect Biology, Ganzhou, China Ganzhou Key Laboratory of Nanling Insect Biology Ganzhou China; 4 none, Northfield, MA, United States of America none Northfield, MA United States of America

**Keywords:** gymnosperm, leafminer, host plant, Pinaceae, Cupressaceae

## Abstract

The leafminers on gymnosperms receive much less attention than those on either angiosperms or ferns. Given the distinctly different leaf shape and leaf venation found in gymnosperms, they would be expected to host significantly different leafminer groups. Very few comprehensive reports on gymnosperm-feeding leafminers have been presented.

Based on the well-studied fauna in North America and Europe, we compiled a list of 133 species, 30 genera and 13 families of gymnosperm-feeding leafminers. The gymnosperm-mining families (in descending order of leafminer number) included Tortricidae, Gelechiidae, Argyresthiidae, Yponomeutidae, Batrachedridae, Pyralidae, Adelidae, Agromyzidae, Blastobasidae, Bucculatricidae, Coleophoridae, Curculionidae and Noctuidae. There were 109 species, 22 genera and ten families in North America and 34 species, 19 genera and nine families in Europe. We compiled a list of 102 species and 16 genera of host plants, belonging to four families: Pinaceae, Cupressaceae, Taxaceae and Zamiaceae. There were 84 host species, 15 genera and three host families in North America and 46 host species, ten genera and three host families in Europe. Dominant gymnosperm-mining families and dominant host families were generally the same in the two continents.

## Introduction

Leafminers are insects that feed inside plant leaves during some or all larval stages, leaving externally visible feeding trails known as leaf mines (Hering 1951, Hespenheide 1991, Dai et al. 2011, Liu et al. 2015, Dai et al. 2018, Yang et al. 2021, Eiseman 2022, Ellis 2022). There are four orders, over 50 families and over 10000 leafminer species worldwide (Csóka 2003, Hirowatari 2009, Liu et al. 2015). Most leafminers are monophagous or oligophagous and different leaf-mining genera or families often have distinct leaf-mine characteristics, which can be helpful for the identification of leafminers (Sinclair and Hughes 2010, Dai et al. 2011, Liu et al. 2015). Moreover, the long-term persistence of leaf mines on either extant or extinct plants can provide a valuable basis for ecologists and paleoecologists to reconstruct life histories and interspecific relationships (Dai et al. 2018, Yang et al. 2021).

Host plants of leafminers encompass nearly all vascular plant groups, including lycophytes, ferns, gymnosperms and angiosperms (Kristensen and Schmidt-Rhaesa 1998, Yang et al. 2021, Eiseman 2022). Unlike the leaves of other plant groups, those of most gymnosperms are needle-like or scale-like, with small leaf areas (Zhang and Gao 2012, Mou et al. 2016, Jacobsen 2021). In addition, leaf veins of many gymnosperms extend to the leaf tips as parallel straight lines and leaf veins of many angiosperms are present as reticulated structures (Boyce 2005, Nicotra et al. 2011). Furthermore, gymnosperms generally have much lower leaf vein densities than angiosperms (Boyce 2008, Boyce 2009, Brodribb and Feild 2010, Brodribb et al. 2010, Nicotra et al. 2011). Small leaves are less likely to be mined than large leaves (Faeth 1991, Low et al. 2009, Dai et al. 2011) and leaf venation patterns may affect the host plant preference and larval feeding paths of leafminers (Ayabe 2010, Dai et al. 2011). Therefore, the leafminers on gymnosperms should be significantly different from those on either typical ferns or angiosperms. Differences between the leafminer faunas of gymnosperms and angiosperms are likely also strongly influenced by phytochemicals, such as secondary metabolites (SMs) (Hespenheide 1991, Dai et al. 2011). Alkaloids, steroids and phenolic acids are less widely distributed in gymnosperms than in angiosperms , while phenypropanoids, lignin and coumarin are more widely distributed in gymnosperms than in angiosperms (Wang 2006, Zhang 2018, Zhang et al. 2020). However, whether these physical and chemical differences shape distinct leafminer groups on gymnosperms and angiosperms requires further investigation.

Compared to angiosperms and ferns (Godfray 1984, Yang et al. 2021), few comprehensive reports on gymnosperm-feeding leafminers have been presented (Eiseman 2022). Therefore, in this study, we compile a checklist of gymnosperm-feeding leafminers and their host plants in North America and Europe, hoping to provide some valuable information on insect-gymnosperm relationships.

## Material and methods

Gymnosperm leafminers and their host plants have been more thoroughly studied in North America and Europe than on other continents (Eiseman 2022, Ellis 2022). The information on names and hosts of gymnosperm-feeding leafminers (Suppl. materials 1, 2) was obtained from the book 'Leafminers of North America' (Eiseman 2022), the websites 'Plant Parasites of Europe' (https://bladmineerders.nl) (Ellis 2022) and 'British Leafminers' (http://www.leafmines.co.uk/index.htm) (Edmunds et al. 2022) and a recent publication on conifer-feeding *Batrachedra* species (Berggren et al. 2022). The number of species in each gymnosperm family and the scientific names, authorships and publication years for insect species were compiled from the website 'Catalogue of Life' (https://www.catalogueoflife.org/) (Hobern et al. 2021).

Note that the book 'Leafminers of North America' covers only miners that occur in the continental US and Canada (Eiseman 2022), so some species that occur only in Mexico or the Caribbean may be missed. The website 'Plant Parasites of Europe' includes records from ornamental and crop plants, but excludes those from indoor ornamentals, greenhouse plants, uncommon ornamentals, exotics in botanical gardens and cultivars (Ellis 2022). Some host plants have been recorded only at the genus level, especially in the European datasets (Edmunds et al. 2022, Eiseman 2022, Ellis 2022). To be consistent in these cases, we use "spp." after a genus name to indicate one or more unknown host plant species in this genus. However, such cases are uncommon in the above sources.

In our checklist, both non-native leafminers and non-native host plants for the respective biogeographical regions are included. For example, *Thuja* and *Pseudotsuga* are not native to Europe, but they were also incorporated into the host plants of European gymnosperm-feeding leafminers and *Spilonotalaricana*, a European moth introduced in North America, is listed for both continents (Eiseman 2022, Ellis 2022). The insect species that feed as leafminers only for part of their larval development (e.g. *Coleotechnitescarbonaria*, *C.gibsonella*) are also included (Eiseman 2022, Ellis 2022). Insects, whose status as leafminers is uncertain and plants that require confirmation as hosts for a given leafminer, are marked with a question mark (?) before their scientific name in Table 1 and Suppl. materials 1, 2. Such insects and plants are included in the statistics. However, insect species without full scientific names (i.e. undescribed species) and those without definite host records to at least the plant genus level (e.g. *Rhyacioniablanchardi*, *R.pallifasciata*, *R.salmonicolor* and *R.versicolor*) are excluded (Eiseman 2022). Such cases are uncommon in the above sources.

There are several parasitic modes of gymnosperm-feeding insects, including miner, borer, galler etc. (Brown 2018, Eiseman 2022, Ellis 2022). Species with parasitic modes, other than leaf-mining, were not included in this study, unless they feed as leafminers in early instars. Bark-mining species (e.g. *Spulerinacorticicola* (*De Prins and De Prins 2022*), *Marmarafasciella*, *M.oregonensis* and other undetermined *Marmara* species (Fitzgerald 1975, Eiseman 2022), were excluded from this study, as were species with unknown parasitic modes.

## Results

In total, there were 133 species, 30 genera and 13 families of gymnosperm-feeding leafminers in the two continents, with 109 species, 22 genera and ten families in North America and 34 species, 19 genera and nine families in Europe, with only ten leafminer species occurring in both North America and Europe (Table 1). The leaf-mining families were Tortricidae, Gelechiidae, Argyresthiidae, Yponomeutidae, Batrachedridae, Pyralidae, Adelidae, Agromyzidae, Blastobasidae, Bucculatricidae, Coleophoridae, Curculionidae and Noctuidae (Table 1).

In Tortricidae, there were 53 species and 12 genera of gymnosperm-feeding leafminers with 64 host plant species and nine host genera, including 43 leafminer species with 58 host species, eight host genera and two host families in North America and 12 leafminer species with 13 host species, six host genera and two host families in Europe (Table 2). Amongst tortricid genera, *Rhyacionia* and *EpinotiaEpinotia Epinotia* were the two richest in gymnosperm-feeding leafminer species. There were 19 leafminer species and 21 host species in *Rhyacionia* and 13 leafminer species and 19 host species in *Epinotia*. Whereas *EpinotiaEpinotia Epinotia* was recorded in both North America and Europe, *Rhyacionia* was recorded only in North America. Amongst tortricid species, *Archipspackardiana*, with 15 host species, was the leafminer with the most host species and was recorded only in North America (Table 1). *Archipsoporana*, with six host species, was the leafminer with the second most host species and was recorded only in Europe (Table 1).

In Gelechiidae, there were 42 species and four genera of gymnosperm-feeding leafminers with 50 host plant species and ten host genera, including 41 leafminer species with 45 host species, ten host genera and two host families in North America and four leafminer species with ten host species, five host genera and two host families in Europe (Table 2). Amongst gelechiid genera, *Coleotechnites* is the richest in gymnosperm-feeding leafminer species, with 33 leafminer species in North America, but only one species in Europe.

In Argyresthiidae, there were 20 species and one genus of gymnosperm-feeding leafminers with 28 host plant species and eight host genera, including 14 leafminer species with 13 host species, seven host genera and two host families in North America and eight leafminer species with 22 host species, five host genera and two host families in Europe (Table 2). All of these insects belong to the genus *Argyresthia* (Table 2).

In Yponomeutidae, there were seven species and three genera of gymnosperm-feeding leafminers with 22 host plant species and one host genus, including four leafminer species with 17 host species, one host genus and one host family in North America and four leafminer species with seven host species, one host genus and one host family in Europe (Table 2). Amongst yponomeutid genera, *Ocnerostoma* is the richest in gymnosperm-feeding leafminer species, with four leafminer species and seven host species.

Of all these leafminers, just one polyphagous species (*Liriomyzaschmidti*) is presumed to feed on both gymnosperms and angiosperms and it is also the only gymnosperm-mining dipteran fly in our study. The other 132 species (i.e. > 99%) feed exclusively on gymnosperms.

For host plants, there were 102 species, 16 genera and four families represented in the two continents. Eighty-four species, 15 genera and three families were found in North America, including Pinaceae, Cupressaceae and Zamiaceae (Table 3). Forty-six species, ten genera and three families were found in Europe, including Pinaceae, Cupressaceae and Taxaceae (Table 3).

In Pinaceae, there were 74 host species, six host genera and 102 associated leafminer species, including 65 host species, six host genera and 83 leafminer species in North America and 29 host species, five host genera and 26 leafminer species in Europe (Table 3). Amongst gymnosperm genera, *Pinus* was richest in both host species and leafminer species, with 38 host species and 67 leafminer species, including 35 host species and 59 leafminer species in North America and eight host species and ten leafminer species in Europe.

In Cupressaceae, there were 26 host species, eight host genera and 32 associated leafminer species, including 18 host species, eight host genera and 25 leafminer species in North America and 16 host species, four host genera and ten leafminer species in Europe (Table 3). Amongst gymnosperm genera, *Juniperus* was second richest in host species and fourth richest in leafminer species, with 14 host species and 22 leafminer species, including nine host species and 15 leafminer species in North America and nine host species and nine leafminer species in Europe (Table 3) .

In North America, gymnosperm-feeding leafminers belong to ten families, mostly in Tortricidae (43 species, 39.45%), Gelechiidae (41 species, 37.61%), Argyresthiidae (14 species, 12.84%), Yponomeutidae (four species, 3.67%) and Pyralidae (two species, 1.83%) (Table 2, Fig. 1a). In Europe, gymnosperm-feeding leafminers belong to nine families, mostly in Tortricidae (12 species, 35.29%), Argyresthiidae (eight species, 23.53%), Gelechiidae (four species, 11.76%), Yponomeutidae (four species, 11.76%) and Batrachedridae (two species, 5.88%) (Table 2, Fig. 1b).

There were two orders and two classes of host plants for gymnosperm-feeding leafminers in the two continents, including the orders of Pinales (class Pinopsida) and Cycadales (class Cycadopsida) (Table 3). Almost all host plants belong to the order Pinales, with just one belonging to the order Cycadales (Table 3).

In North America, there were three families and two orders of host plants, including Pinales (Pinaceae and Cupressaceae) and Cycadales (Zamiaceae) (Table 3). In Europe, there were three families of host plants, all belonging to the order Pinales, including Pinaceae, Cupressaceae and Taxaceae (Table 3). Pinaceae had the largest number of host species in both North America and Europe, but with different genera having the most host species. In North America, the genus *Pinus* (35 host species) of Pinaceae had the most host species (Table 3), while the genera *Abies* (nine host species) of Pinaceae and *Juniperus* (nine host species) of Cupressaceae had the most host species in Europe (Table 3).

## Discussion

In this paper, we provide a list of the known gymnosperm-feeding leafminers and their host plants in North America and Europe. Here, we give a brief overiew of gymnosperm-mining families in the two continents, in descending order of species richness:

(1) Tortricidae (junior synonym: Olethreutidae) (Lepidoptera). Insects within this family are able to feed on many plant parts, usually as leaf-rollers or borers, with some inducing galls (Brown 2018, Eiseman 2022, Ellis 2022). Only a few tortricid species have mining behaviour (Ellis 2022). Most of these are not typical leafminers, with the mining behaviour occurring only in early instars and older larvae feed as borers in other plant parts or externally in leaf shelters (Eiseman 2022, Ellis 2022). It should be noted here that species of *Rhyacionia*, which we have reported to be the tortricid genus richest in gymnosperm leaf-mining species, feed primarily as shoot borers. The few *Rhyacionia* species for which detailed life history information is available all feed initially as leafminers and the rest are only assumed to do so (Eiseman 2022). On the other hand, several *Epinotia* species do mine in leaves of Pinaceae throughout their development (Eiseman 2022). Many tortricid species are conifer-feeding specialists (Brown 2018). For example, the leaf-mining species *Choristoneurafumiferana* feeds exclusively on Pinaceae (Eiseman 2022) and is an important pest of coniferous forest trees in North America (Brown 2018). The number of leafminer species in this family was greatest amongst all mining families of gymnosperms in both North America and Europe (Table 2).

(2) Gelechiidae (Lepidoptera). Host plants of this family are extremely diverse, with more than 80 host families utilised (Kristensen and Schmidt-Rhaesa 1998). Only a small proportion of gelechiid species have mining behaviour (Kristensen and Schmidt-Rhaesa 1998) and, as with Tortricidae, many of them do so only in early instars (Eiseman 2022). The larvae of several gelechiid genera feed on gymnosperms and three of these (*Coleotechnites*, *Exoteleia* and *Chionodes*) include species that feed exclusively on Pinaceae (Brown 2018). One needle-mining *Coleotechnites* species was first observed on *Pinusjeffreyi* in southern California in 1963, but remains undescribed (Luck 1976, Eiseman 2022).

(3) Argyresthiidae (Lepidoptera). This family includes about 160 species worldwide (Eiseman 2022). The larvae usually feed on host plants as miners or as borers in buds, flowers, seeds, fruit, cones or twigs (Eiseman 2022). In total, 42% of species feed on conifers as leafminers (Powell 1980, Brown 2018). Over 13 families of plants are utilised by larvae of the genus *Argyresthia* (gymnosperms and dicots) (Kristensen and Schmidt-Rhaesa 1998). However, there is no record of larvae of this genus feeding on angiosperms as leafminers, with the exception of an erroneous report of the willow-feeding species *A.pygmaeella* doing so (it is in fact a shoot- and catkin-borer) (Eiseman 2022).

(4) Yponomeutidae (Lepidoptera). At least 18 plant families are utilised by yponomeutid moths, including gymnosperms (Pinaceae) and angiosperms (e.g. Betulaceae, Celastraceae and Rosaceae) (Ulenberg 2009). Most yponomeutid species are external feeders on leaves (Kristensen and Schmidt-Rhaesa 1998) and only a few are leafminers (Eiseman 2022). The larvae of three yponomeutid genera feed on gymnosperms (Eiseman 2022, Ellis 2022). Amongst the pine needle-mining *Ocnerostoma* species is one that feeds on *Pinusresinosa* and remains undescribed (Freeman 1960, Maier et al. 2011). *Zelleriahaimbachi* is another *Pinus*-mining specialist (Brown 2018, Eiseman 2022); when nearly mature, the larvae move more freely and web around the needle bases (Eiseman 2022).

(5) Batrachedridae (Lepidoptera). This is a small family of tiny moths, with only a few species feeding as leafminers (Ellis 2022). In North America, just two species are known to feed as leafminers (*Batrachedraconcitata* on *Agave* and *B.pinicolella* on *Abies*) (Eiseman 2022). Two gymnosperm-mining batrachedrid species occur in Europe and one of these (*B.pinicolella*) has recently invaded North Amercia (Maier 2005, Berggren et al. 2022).

(6) Pyralidae (Lepidoptera). The larvae of pyralid moths have diverse types of feeding behaviour. Some act as leaf-tiers or leaf-rollers, others as borers in stems, cambium or fruit and still others even feed on dead plant material (Eiseman 2022, Ellis 2022). Only a few pyralid species are leafminers, with very few feeding on gymnosperms (Eiseman 2022, Ellis 2022). In North America and Europe, just two species are gymnosperm-feeding leafminers (*Pococerarobustella* and *Dioryctriareniculelloides*), with both mining leaves only in early instars (Eiseman 2022, Ellis 2022).

(7) Adelidae (Lepidoptera). This is a small and relatively primitive family (Kozlov and Robinson 1996) with about 350 species (Hobern et al. 2021). Many adelid species are leafminers in their early instars (Kozlov and Robinson 1996). Just one species (*Nemophoraassociatella*) is recorded as an *Abies*-feeding leafminer in Europe (Ellis 2022).

(8) Agromyzidae (Diptera). This is a family widely distributed throughout the world (Mujica and Kroschel 2011) and it is one of the largest fly families (Civelek 2003, Dousti 2010). It is well-known for diverse leafminers, some of which are economically important (Benavent-Corai et al. 2005). About half of agromyzid species are leafminers (Ellis 2022). The genus *Liriomyza* alone has more than 300 leaf-mining species (Mujica and Kroschel 2011). More than ten agromyzid genera have mining behaviour in North America alone, but just one species is known to be a gymnosperm-feeding leafminer (Eiseman 2022). *Liriomyzaschmidti* is an extremely polyphagous species with reported hosts in nearly 30 families, one of which is the cycad family Zamiaceae (Eiseman 2022). No agromyzid species is recorded as a gymnosperm-feeding leafminer in Europe (Ellis 2022).

(9) Blastobasidae (Lepidoptera). This is a relatively small moth family with a wide distribution around the world (Karsholt and Sinev 2004, Ellis 2022). Most blastobasid larvae are detritus feeders (Ellis 2022). Conifer-feeding blastobasids are cone feeders or external webbers (Brown 2018) and there are six pine-feeding specialists in the genus *Holcocera* (Brown 2018). Just one blastobasid species (*Blastobasisvittata*) is a gymnosperm leafminer in Europe, occasionally mining in yew (*Taxusbaccata*) or spruce (*Picea* sp.) as young larvae (Ellis 2022).

(10) Bucculatricidae (Lepidoptera). This family has a wide distribution around the world (Vargas and Mundaca 2016, Tokár and Laštůvka 2018). Most bucculatricid species are leafminers in their early instars, while a few species are gallers (Vargas and Mundaca 2016). *Bucculatrixinusitata* is reportedly a Cupressaceae specialist (Brown 2018), but this is based on a single specimen that is labelled as having been reared from a larva on *Juniperus* (Braun 1963). This host record requires confirmation, since *Bucculatrix* larvae commonly wander from their food plants before spinning cocoons and *B.inusitata* belongs to a group of species that otherwise are virtually all Asteraceae specialists (Braun 1963). No other Bucculatricidae have been reported from gymnosperms in North America or Europe (Eiseman 2022, Ellis 2022).

(11) Coleophoridae (Lepidoptera). Virtually all coleophorid larvae begin their lives as leafminers or within ovules and seeds and, at later larval stages, live in portable silk cases, from which the leaf-mining species continue to make fleck mines (Eiseman 2022, Ellis 2022). In North America and Europe, there is just one gymnosperm-mining species (*Coleophoralaricella*), which is native to Europe and was introduced in North America in the 1800s (Eiseman 2022, Ellis 2022).

(12) Curculionidae (Coleoptera). This family has a wide distribution around the world (Wikipedia 2022) and it is the largest beetle family (Bandeira et al. 2021), but only a small proportion of curculionid species are leafminers (Ellis 2022). Thirty-one species and eight genera in Curculionidae are leafminers in North America, none of them feeding on gymnosperms (Eiseman 2022). However, one curculionid species (*Brachonyxpineti*) has been recorded as a gymnosperm-feeding leafminer in Europe (Ellis 2022).

(13) Noctuidae (Lepidoptera). This is amongst the largest moth families (Ellis 2022). Many noctuid larvae are external feeders or borers in either stems or roots (Eiseman 2022, Ellis 2022), while only several species have mining behaviour (Eiseman 2022). First instars of a single North American species (*Feraliajocosa*) have been observed to mine into *Tsuga* needles, although they do not fully enter the needles as with typical miners (Eiseman 2022). No noctuid species mine gymnosperms in Europe (Ellis 2022).

In both North America and Europe, leafminer groups on angiosperms are extraordinarily diverse, followed by those on gymnosperms, while those on ferns and their allies are the least (Suppl. material 3; Yang et al. 2021, Eiseman 2022, Ellis 2022). Amongst the leafminers on either ferns or angiosperms, Lepidoptera has the most leaf-mining families (Yang et al. 2021, Eiseman 2022, Ellis 2022). Similarly, 11 of the 13 gymnosperm-feeding families belong to Lepidoptera (Table 2). In contrast with angiosperms and ferns, gymnosperms host just one leaf-mining beetle (Coleoptera), one leaf-mining fly (Diptera) and no leaf-mining sawfly (Hymenoptera) species (Table 1). Several leafminer families such as Noctuidae, Curculionidae and Agromyzidae occur in all three vascular plant groups (Yang et al. 2021, Eiseman 2022, Ellis 2022). Agromyzidae is a family best known as leafminers (Spencer 1990). Curculionidae has many leafminer species, but Noctuidae has only a few (Eiseman 2022). Whereas ferns and angiosperms host many specialised leafmining families (e.g. Gracillariidae, Agromyzidae, Anthomyiidae, Tischeriidae, Acanthopteroctetidae, Nepticulidae, Heliozelidae, Bedelliidae, Lyonetiidae, Elachistidae) (Kristensen and Schmidt-Rhaesa 1998, Yang et al. 2021, Eiseman 2022), virtually none of the leafminers of gymnosperms belongs to specialised leafmining families, with the exception of a single extremely polyphagous agromyzid that is believed to be responsible for mines found on Cycadales. It is worth mentioning that, although there are no leafmining gracillariid species on gymnosperms in North America or Europe, there are bark-miners in the genus *Marmara* (e.g. *M.fasciella* and *M.oregonensis*) (Eiseman 2022). However, the focus of this study is on leaf-mining species.

Some insect families, known for their leafmining habits, are absent or rare on gymnosperms (e.g. Tischeriidae, Nepticulidae, Gracillariidae and Agromyzidae). Worldwide, no species of Tischeriidae (Kristensen and Schmidt-Rhaesa 1998, Xu et al. 2021, Alipanah et al. 2022) or Nepticulidae (Powell 1980, Kristensen and Schmidt-Rhaesa 1998, Menken et al. 2009, Doorenweerd et al. 2015) are recorded as leafminers on gymnosperms; both families occur only on angiosperms. In Gracillariidae, although most larvae are leafminers (at least during their early instars) (Eiseman 2022, Ellis 2022), no gracillariid leafminer is recorded on gymnosperms in either North America or Europe (Suppl. material 3; Eiseman 2022, Ellis 2022). However, two gracillariid leafminers are known to feed on gymnosperms in other regions (see below) (Liu et al. 2018, De Prins and De Prins 2022). Agromyzidae, another well-known leafmining family, has many leafminer species on angiosperms, ferns and even bryophytes, but only *Liriomyzaschmidti* is recorded from a gymnosperm and this association has yet to be confirmed by rearing (Spencer 1990, Yang et al. 2021, Eiseman 2022, Ellis 2022).

Generally, North America is richer in gymnosperm leafminers than Europe (species 109:34; genus 22:19; family 10:9) (Table 2). The higher diversity of leafminers found in North America is probably correlated with higher availability of potential host plants. North America has a larger species number and distribution area of gymnosperm hosts than Europe (Fragnière et al. 2015). For example, cycads exist in the Rocky Mountain and Caribbean regions in North America, but not in Europe (Fragnière et al. 2015). In North America, gnetophytes are distributed in the Caribbean regions, Mexico and the vast regions of the US, while in Europe, there are just a few distributed near the southern coast (Fragnière et al. 2015). Although conifers occur in almost all regions of North America (ca. 330 species) and Europe (ca. 100 species), their species number and distribution area in North America is larger (Flora of North America Editorial Committee 1993, Euro+Med 2006, Fragnière et al. 2015).

Leaf-mining insects are greatly affected by leaf features of host plants, including leaf size, leaf thickness, leaf venation and leaf phytochemicals (Hespenheide 1991, Dai et al. 2011). Differences in these features may explain why gymnosperms and angiosperms have significantly different leafminer species. On the other hand, gymnosperms with leaf features similar to those of angiosperms may have correspondingly similar leafminer groups. *Gnetum* (Gymnospermae: Gnetaceae) is a representative example, with a leaf type closely approximate to that of angiosperms (Spencer 1990, Stewart et al. 1993). Therefore, it is a suitable host candidate for Agromyzidae (Spencer 1990), which is a common leafminer family on angiosperms. Cycads likewise share some herbivorous insect species with angiosperms (Whitaker and Salzman 2020). The palm-like leaves of the cycad *Zamiaintegrifolia* (Zamiaceae) might explain why it is an acceptable host for the polyphagous species *Liriomyzaschmidti*, which otherwise is only known to feed on angiosperms (Eiseman 2022). Gracillariidae is a well-known leafminer family, but just two species have been found to feed in leaves of gymnosperms (the Japanese *Phyllocnistispodocarpa* mines in *Podocarpusmacrophyllus* (Podocarpaceae) and in New Zealand *Parectopaleucocyma* mines in *Agathisaustralis* (Araucariaceae)) (Liu et al. 2018, De Prins and De Prins 2022). This may also be related to the unusual leaf morphology of the host plants. Unlike most conifer species with leaves in the form of scales or needles, podocarps have larger, bilaterally flattened leaves and vein reticulation (Brodribb 2011). Similarly, compared with other typical gymnosperms, the leaves of the genus *Agathis* are more broad and flattened with rounded tips (Stockey 1982). Therefore, the distinctive leaf structure of host plants may cause the gracillariid leafminers to mine selectively on gymnosperms. Podocarpaceae and Araucariaceae are sister families (Quinn et al. 2002), implying that plant phylogeny and phytochemistry might also play a role in determining the presence/absence of leafminers on plants.

## Supplementary Material

3BC8B84C-6456-50CA-AD55-42AA15E9F1C9 10.3897/BDJ.10.e91313.suppl1 Supplementary material 1 The gymnosperm-feeding leafminers and their host plants in North America Data type Number of species File: oo_747747.xlsx https://binary.pensoft.net/file/747747 Taibin Chen, Xiaohua Dai, Charles Eiseman

E1A8D5AD-AFAB-51C1-8D23-14EA874C1D90 10.3897/BDJ.10.e91313.suppl2 Supplementary material 2 The gymnosperm-feeding leafminers and their host plants in Europe Data type Number of species File: oo_747748.xlsx https://binary.pensoft.net/file/747748 Taibin Chen, Xiaohua Dai, Charles Eiseman

EE559F3F-65BB-5D1D-ABBB-27351D2A5ABA 10.3897/BDJ.10.e91313.suppl3 Supplementary material 3 Insect families of leafminers on ferns, gymnosperms, angiosperms in North America and Europe Data type Insect families File: oo_747030.docx https://binary.pensoft.net/file/747030 Taibin Chen, Xiaohua Dai, Charles Eiseman

## Figures and Tables

**Figure 1a.**
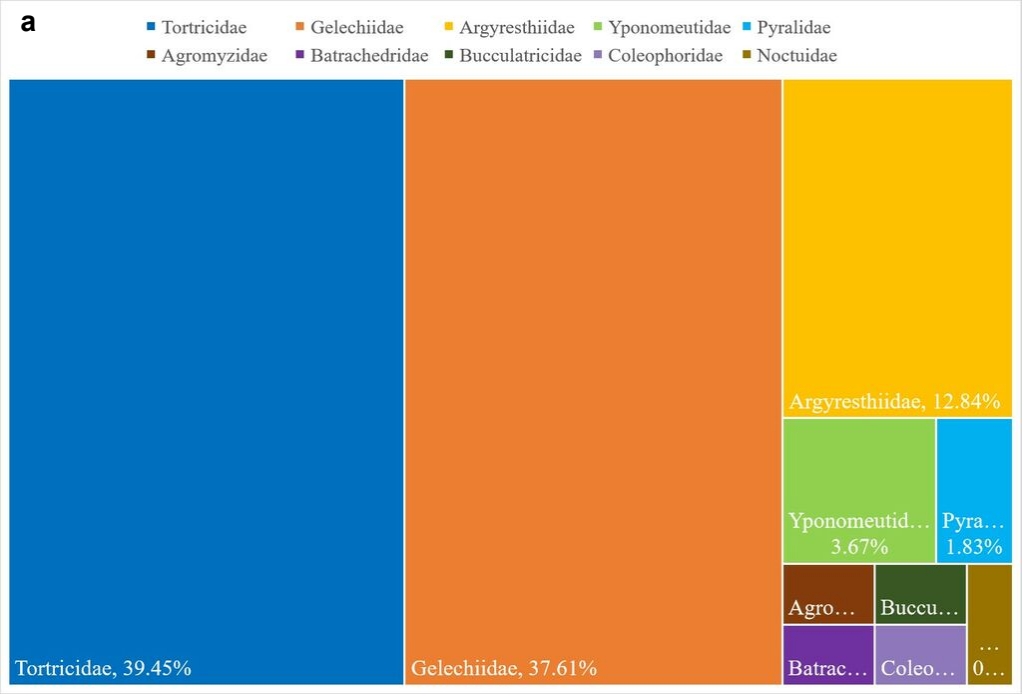
North America

**Figure 1b.**
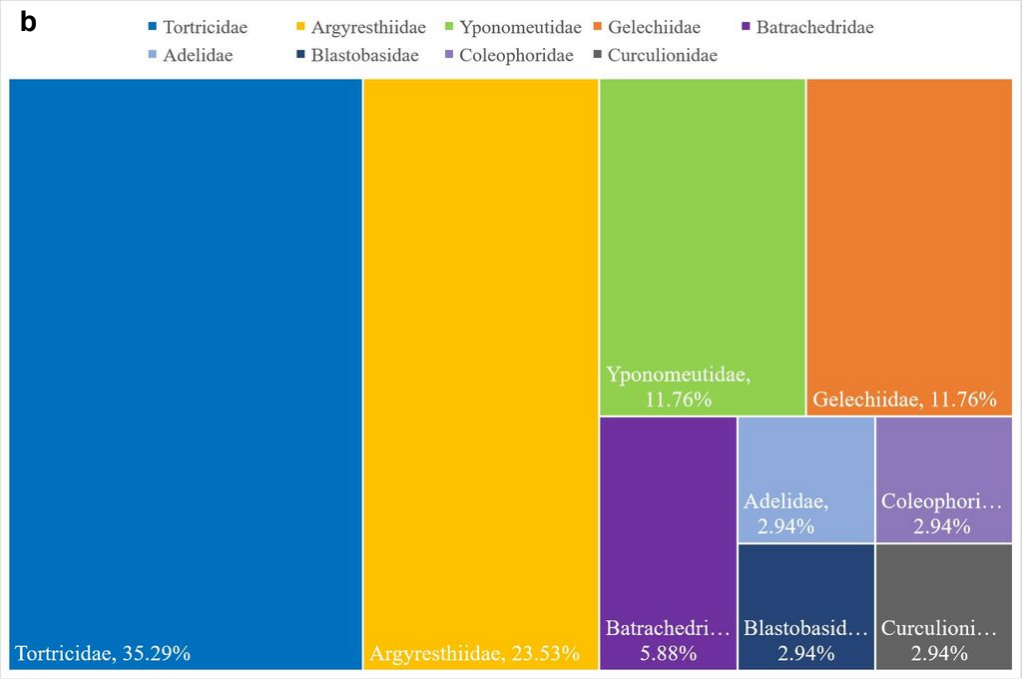
Europe

**Table 1.** A checklist of gymnosperm-feeding leafminers in North America and Europe.

**Miner family**	**Miner species**	**Host plants**	**Distribution Region**
Lepidoptera			
Adelidae	*Nemophoraassociatella* Zeller, 1839	*Abiesalba*	Europe
Argyresthiidae	*Argyresthiaabdominalis* Zeller, 1839	*Juniperuscommunis*	Europe
Argyresthiidae	*Argyresthiaaffinis* Braun, 1940	*Juniperusvirginiana*	North America
Argyresthiidae	*Argyresthiaannettella* Busck, 1907	*Juniperuscommunis*	North America
Argyresthiidae	*Argyresthiaarceuthobiella* Busck, 1917	*Calocedrusdecurrens*	North America
Argyresthiidae	*Argyresthiaaureoargentella* Brower, 1953	*Thujaoccidentalis*	North America
Argyresthiidae	*Argyresthiaaurulentella* Stainton, 1849	*Juniperuscommunis*, *Juniperusfoetidissima*	Europe
Argyresthiidae	*Argyresthiacanadensis* Freeman, 1972	*Thujaoccidentalis*	North America
Argyresthiidae	*Argyresthiacupressella* Walsingham, 1890	*Chamaecyparis* spp., *Cupressus* spp., *Juniperus* spp., *Sequoiasempervirens*, *Thuja* spp.	North America, Europe*
Argyresthiidae	*Argyresthiadilectella* Zeller, 1874	*Chamaecyparis* spp., *Juniperuscommunis*, *Juniperussabina*	Europe
Argyresthiidae	*Argyresthiaflexilis* Freeman, 1960	*Pinusflexilis*	North America
Argyresthiidae	*Argyresthiafranciscella* Busck, 1915	*Cupressusmacrocarpa*	North America
Argyresthiidae	*Argyresthiafreyella* Walsingham, 1890	*Juniperusvirginiana*	North America
Argyresthiidae	*Argyresthiafundella* Fischer von Röslerstamm, 1835	*Abiesalba*, *Abiesbalsamea*, *Abiesconcolor*, *Abiesgrandis*, *Abiesnordmanniana*, *Abiesnumidica*	Europe
Argyresthiidae	*Argyresthialibocedrella* Busck, 1917	*Calocedrusdecurrens*, *Chamaecyparislawsoniana*	North America
Argyresthiidae	*Argyresthiapilatella* Braun, 1910	*Pinusradiata*, ?*Pinustorreyana*	North America
Argyresthiidae	*Argyresthiareticulata* Staudinger, 1877	*Juniperuscommunis*	Europe
Argyresthiidae	?*Argyresthiathoracella* Busck, 1907	*Juniperus* spp.	North America
Argyresthiidae	*Argyresthiathuiella* Packard, 1871	*Chamaecyparislawsoniana*, *Cupressus* spp.,*Thujaoccidentalis*, *Thujaplicata*	North America, Europe*
Argyresthiidae	*Argyresthiatrifasciae* Braun, 1910	*Cupressusmacrocarpa*	North America
Argyresthiidae	*Argyresthiatrifasciata* Staudinger, 1871	*Chamaecyparislawsoniana*, *Cupressus* x *leylandii*, *Juniperuschinensis*, *Juniperushorizontalis*, *Juniperussabina*, *Juniperussquamata*, *Juniperusvirginiana*, *Juniperus* x *media*, *Thujaoccidentalis*	Europe
Batrachedridae	*Batrachedraconfusella* Berggren, Aarvik, Huemer, Lee et Mutanen, 2022	*Pinussylvestris*	Europe
Batrachedridae	*Batrachedrapinicolella* Zeller, 1839	*Abies* spp., *Piceaabies*, *Piceaglauca*, *Piceapungens*, *Picearubens*, *Pinus* spp.	North America*, Europe
Blastobasidae	*Blastobasisvittata* Wollaston, 1858	*Picea* spp., *Taxusbaccata*	Europe
Bucculatricidae	*Bucculatrixinusitata* Braun, 1963	?*Juniperuscommunis*	North America
Coleophoridae	*Coleophoralaricella* Hübner, 1817	*Larixdecidua*, *Larixgmelinii*, *Larixkaempferi*, *Larixlaricina*, *Larixoccidentalis*, *Larixsibirica*, *Pseudotsugamenziesii*	North America*, Europe
Gelechiidae	*Chionodeselectella* Zeller, 1839	*Abiesalba*, *Abiespinsapo*, *Juniperuscommunis*, *Piceaabies*	Europe
Gelechiidae	*Chionodesretiniella* Barnes & Busck, 1920	*Pinusponderosa*, *Pinussabiniana*, *Tsugaheterophylla*	North America
Gelechiidae	*Coleotechnitesalbicostata* Freeman, 1965	*Juniperusvirginiana*	North America
Gelechiidae	*Coleotechnitesapicitripunctella* Clemens, 1860	*Tsugacanadensis*	North America
Gelechiidae	*Coleotechnitesardas* Freeman, 1960	Pinuscontortavar.latifolia	North America
Gelechiidae	*Coleotechnitesatrupictella* Dietz, 1900	*Abiesbalsamea*, *Abiesgrandis*, *Abieslasiocarpa*, *Piceaengelmannii*, *Piceaglauca*, *Piceamariana*, *Picearubens*, *Piceasitchensis*, *Pinusponderosa*, *Pseudotsugamenziesii*, *Tsugaheterophylla*	North America
Gelechiidae	*Coleotechnitesbiopes* Freeman, 1960	Pinuscontortavar.latifolia	North America
Gelechiidae	*Coleotechnitesblastovora* McLeod, 1962	*Piceaglauca*	North America
Gelechiidae	*Coleotechnitescanusella* Freeman, 1957	*Pinusbanksiana*, Pinuscontortavar.latifolia	North America
Gelechiidae	*Coleotechnitescarbonaria* Freeman, 1965	*Juniperus* spp.	North America
Gelechiidae	*Coleotechnitescondignella* Busck, 1929	*Pinusponderosa*	North America
Gelechiidae	*Coleotechnitesconiferella* Kearfott, 1907	*Pinusbanksiana*	North America
Gelechiidae	*Coleotechnitesducharmei* Freeman, 1962	*Piceaglauca*, *Piceamariana*, *Picearubens*	North America
Gelechiidae	*Coleotechnitesedulicola* Hodges & Stevens, 1978	*Pinusedulis*	North America
Gelechiidae	*Coleotechnitesflorae* Freeman, 1960	Pinuscontortavar.latifolia	North America
Gelechiidae	*Coleotechnitesgibsonella* Kearfott, 1907	*Juniperuscommunis*	North America
Gelechiidae	*Coleotechnitesgranti* Freeman, 1965	*Abiesgrandis*	North America
Gelechiidae	*Coleotechnitesjuniperella* Kearfott, 1903	*Juniperuscommunis*	North America
Gelechiidae	*Coleotechniteslaricis* Freeman, 1965	*Larixlaricina*	North America
Gelechiidae	*Coleotechniteslewisi* Freeman, 1960	*Pinusflexilis*	North America
Gelechiidae	*Coleotechnitesmacleodi* Freeman, 1965	*Tsugacanadensis*	North America
Gelechiidae	*Coleotechnitesmartini* Freeman, 1965	*Piceaabies*, *Piceaglauca*	North America
Gelechiidae	*Coleotechnitesmilleri* Busck, 1914	Pinuscontortavar.murrayana	North America
Gelechiidae	?*Coleotechnitesmoreonella* Heinrich, 1920	?*Pinusponderosa*, Pinusponderosavar.scopulorum	North America
Gelechiidae	*Coleotechnitesobliquistrigella* Chambers, 1872	*Juniperusvirginiana*	North America
Gelechiidae	*Coleotechnitesoccidentis* Freeman, 1965	*Juniperusscopulorum*	North America
Gelechiidae	*Coleotechnitespiceaella* Kearfott, 1903	*Abiesbalsamea*, *Piceaabies*, *Piceaglauca*, *Piceamariana*, *Piceaomorika*, *Piceapungens*, *Picearubens*	North America, Europe*
Gelechiidae	*Coleotechnitespinella* Busck, 1906	*Pinusponderosa*	North America
Gelechiidae	*Coleotechnitesponderosae* Hodges & Stevens, 1978	*Pinusponderosa*	North America
Gelechiidae	*Coleotechnitesresinosae* Freeman, 1960	*Pinusbanksiana*, *Pinusresinosa*	North America
Gelechiidae	*Coleotechnites* sp.	*Pinusjeffreyi*	North America
Gelechiidae	*Coleotechnitesstanfordia* Keifer, 1933	*Cupressusmacrocarpa*	North America
Gelechiidae	*Coleotechnitesstarki* Freeman, 1957	Pinuscontortavar.latifolia	North America
Gelechiidae	*Coleotechnitesthujaella* Kearfott, 1903	*Thujaoccidentalis*	North America
Gelechiidae	?*Coleotechnitesvariiella* Chambers, 1872	*Taxodiumdistichum*	North America
Gelechiidae	*Dichomerismarginella* Fabricius, 1781	*Juniperus* spp.	North America*, Europe
Gelechiidae	*Exoteleiaanomala* Hodges, 1986	*Pinusponderosa*	North America
Gelechiidae	*Exoteleiaburkei* Keifer, 1932	*Pinusattenuata*, *Pinuscoulteri*, *Pinusradiata*, *Pinussabiniana*	North America
Gelechiidae	*Exoteleiachillcotti* Freeman, 1963	*Pinuspalustris*	North America
Gelechiidae	*Exoteleiadodecella* Linnaeus, 1758	*Larixdecidua*, *Pinusbanksiana*, *Pinusmugo*, *Pinusnigra*, *Pinusresinosa*, *Pinusstrobus*, *Pinussylvestris*, *Pinusuncinata*	North America*, Europe
Gelechiidae	*Exoteleianepheos* Freeman, 1967	*Pinusmugo*, *Pinusresinosa*, *Pinussylvestris*	North America
Gelechiidae	*Exoteleiapinifoliella* Chambers, 1880	*Pinusbanksiana*, *Pinusechinata*, *Pinuspalustris*, *Pinuspungens*, *Pinusresinosa*, *Pinusrigida*, *Pinussylvestris*, *Pinustaeda*, *Pinusvirginiana*, ?*Pinuscontorta*, ?*Pinusponderosa*	North America
Noctuidae	*Feraliajocosa* Guenée, 1852	*Abiesbalsamea*, *Abiesgrandis*, *Abieslasiocarpa*, *Larixlaricina*, *Larixoccidentalis*, *Piceamariana*, *Piceaengelmannii*, *Piceaglauca*, *Piceasitchensis*, *Pseudotsugamenziesii*, *Tsugacanadensis*, *Tsugaheterophylla*, *Tsugamertensiana*	North America
Pyralidae	*Dioryctriareniculelloides* Mutuura & Munroe, 1973	*Abies* spp., *Larix* spp., *Piceaengelmannii*, *Piceaglauca*, *Piceamariana*, *Piceapungens*, *Piceasitchensis*, ?*Picearubens*, ?*Pinuscontorta*, *Pseudotsugamenziesii*, *Tsugaheterophylla*	North America
Pyralidae	*Pococerarobustella* Zeller, 1848	*Pinusbanksiana*, *Pinusechinata*, *Pinuselliottii*, *Pinusmugo*, *Pinuspalustris*, *Pinusresinosa*, *Pinusrigida*, *Pinusstrobus*, *Pinussylvestris*, *Pinustaeda*, *Pinusvirginiana*	North America
Tortricidae	*Aethesrutilana* Hübner, 1817	*Juniperus* spp.	North America
Tortricidae	*Archipsoporana* Linnaeus, 1758	*Abiesalba*, *Juniperuscommunis*, *Larixdecidua*, *Piceaabies*, *Pinussylvestris*, *Thujaoccidentalis*	Europe
Tortricidae	*Archipspackardiana* Fernald, 1886	*Abiesamabilis*, *Abiesbalsamea*, *Abieslasiocarpa*, *Larix* spp., *Piceaabies*, *Piceaengelmannii*, *Piceaglauca*, *Piceamariana*, *Piceapungens*, *Picearubens*, *Piceasitchensis*, *Pinusbanksiana*, *Pinuscontorta*, *Tsugacanadensis*, *Tsugaheterophylla*	North America
Tortricidae	*Argyrotaeniaoccultana* Freeman, 1942	*Abiesbalsamea*, *Larixlaricina*, *Piceaengelmannii*, *Piceaglauca*, *Piceamariana*, *Picearubens*, *Pinuscontorta*, *Pseudotsugamenziesii*, *Tsuga* spp.	North America
Tortricidae	*Argyrotaeniapinatubana* Kearfott, 1905	*Pinusstrobus*	North America
Tortricidae	*Argyrotaeniatabulana* Freeman, 1944	*Pinusalbicaulis*, *Pinusbanksiana*, Pinuscontortavar.latifolia, *Pinusponderosa*	North America
Tortricidae	*Choristoneuracarnana* Barnes & Busck, 1920	*Abiesconcolor*, *Pseudotsugamacrocarpa*, *Pseudotsugamenziesii*	North America
Tortricidae	*Choristoneurafumiferana* Clemens, 1865	*Abiesbalsamea*, *Larixlaricina*, *Piceaglauca*, *Picearubens*, *Pinus* spp.	North America
Tortricidae	*Choristoneurahoustonana* Grote, 1873	*Juniperusashei*, *Juniperuscalifornica*, *Juniperuschinensis*, *Juniperusscopulorum*, *Juniperusvirginiana*, ?*Juniperusoccidentalis*, ?*Juniperusosteosperma*, *Juniperusvirginiana*	North America
Tortricidae	*Choristoneuralambertiana* Busck, 1915	*Abies* spp., *Pinus* spp.	North America
Tortricidae	*Choristoneuraoccidentalis* Freeman, 1967	*Abies* spp., *Picea* spp.	North America
Tortricidae	*Choristoneuraorae* Freeman, 1967	*Abiesamabilis*, *Piceasitchensis*	North America
Tortricidae	*Choristoneurapinus* Freeman, 1953	*Pinus* spp.	North America
Tortricidae	*Choristoneuraretiniana* Walsingham, 1879	*Abiesconcolor*, *Abiesgrandis*, *Abiesmagnifica*	North America
Tortricidae	*Choristoneuraspaldingana* Obraztsov, 1962	*Abiesconcolor*	North America
Tortricidae	*Clavigestapurdeyi* Durrant, 1911	*Pinuscontorta*, *Pinusnigra*, *Pinussylvestris*	Europe
Tortricidae	*Cymolomiahartigiana* Ratzeburg, 1840	*Abiesalba*, *Piceaabies*	Europe
Tortricidae	*Dicheliahistrionana* Frölich, 1828	*Abiesalba*, *Piceaabies*	Europe
Tortricidae	*Epinotiaaridos* Freeman, 1960	Pinuscontortavar.latifolia	North America
Tortricidae	*Epinotiabalsameae* Freeman, 1965	*Abiesbalsamea*	North America
Tortricidae	*Epinotiafraternana* Haworth, 1811	*Abiesalba*, *Abiescephalonica*, *Abiesgrandis*, *Abiesnordmanniana*	Europe
Tortricidae	*Epinotiahopkinsana* Kearfott, 1907	*Piceasitchensis*, *Pinus* spp.	North America
Tortricidae	*Epinotiameritana* Heinrich, 1923	*Abiesconcolor*, *Abiesmagnifica*	North America
Tortricidae	*Epinotiananana* Treitschke, 1835	*Abiesalba*, *Piceaabies*, *Piceaglauca*, *Piceamariana*, *Piceapungens*, *Picearubens*, *Piceasitchensis*	North America*, Europe
Tortricidae	*Epinotianormanana* Kearfott, 1907	*Piceaabies*, *Piceaglauca*, *Piceapungens*, *Picearubens*	North America
Tortricidae	*Epinotiapusillana* Peyerimhoff, 1863	*Abiesalba*	Europe
Tortricidae	*Epinotiapygmaeana* Hübner, 1799	*Abiesalba*, *Piceaabies*, *Piceasitchensis*	Europe
Tortricidae	*Epinotiasubsequana* Haworth, 1811	*Abiesalba*, *Abiesgrandis*, *Piceaabies*	Europe
Tortricidae	*Epinotiatedella* Clerck, 1759	*Piceaabies*	Europe
Tortricidae	*Epinotiatrossulana* Walsingham, 1879	*Abiesconcolor*, *Abiesmagnifica*, ?*Pseudotsugamenziesii*	North America
Tortricidae	*Epinotiatsugana* Freeman, 1967	*Abiesamabilis*, *Piceasitchensis*, *Tsugaheterophylla*, *Tsugamertensiana*	North America
Tortricidae	*Pseudohermeniasabietana* Fabricius, 1787	*Abiesalba*, *Piceaabies*	Europe
Tortricidae	*Rhyacioniaadana* Heinrich, 1923	*Pinusbanksiana*, *Pinusresinosa*, *Pinussylvestris*	North America
Tortricidae	?*Rhyacioniaaktita* Miller, 1978	*Pinuselliottii*, *Pinusrigida*, *Pinustaeda*	North America
Tortricidae	*Rhyacioniabuoliana* Denis & Schiffermüller, 1775	*Pinusbanksiana*, *Pinusdensiflora*, *Pinusmugo*, *Pinusnigra*, *Pinuspalustris*, *Pinusponderosa*, *Pinusresinosa*, *Pinusrigida*, *Pinusstrobus*, *Pinussylvestris*, *Pinusthunbergii*, *Pinusvirginiana*	North America
Tortricidae	?*Rhyacioniabusckana* Heinrich, 1923	*Pinusponderosa*, *Pinusresinosa*, *Pinussylvestris*	North America
Tortricidae	?*Rhyacioniabushnelli* Busck, 1914	*Pinusbanksiana*, *Pinusnigra*, *Pinusponderosa*, *Pinusresinosa*, *Pinussylvestris*	North America
Tortricidae	*Rhyacioniafrustrana* Comstock, 1880	*Pinusechinata*, *Pinuselliottii*, *Pinuspalustris*, *Pinusponderosa*, *Pinusradiata*, *Pinusresinosa*, *Pinusrigida*, *Pinusstrobus*, *Pinussylvestris*, *Pinustaeda*, *Pinusvirginiana*	North America
Tortricidae	?*Rhyacioniafumosana* Powell & Miller, 1978	*Pinusponderosa*	North America
Tortricidae	?*Rhyacioniagranti* Miller, 1985	*Pinusbanksiana*	North America
Tortricidae	?*Rhyacioniajenningsi* Powell, 1978	*Pinusponderosa*	North America
Tortricidae	?*Rhyacioniamartinana* Powell, 1978	?*Pinusedulis*	North America
Tortricidae	?*Rhyacioniamonophylliana* Kearfott, 1907	*Pinusmonophylla*	North America
Tortricidae	?*Rhyacioniamultilineata* Powell, 1978	*Pinusponderosa*	North America
Tortricidae	?*Rhyacionianeomexicana* Dyar, 1903	*Pinusponderosa*	North America
Tortricidae	?*Rhyacioniapasadenana* Kearfott, 1907	*Pinuscontorta*, *Pinusmuricata*, *Pinusradiata*	North America
Tortricidae	?*Rhyacioniarigidana* Fernald, 1880	*Pinusechinata*, *Pinusresinosa*, *Pinusrigida*, *Pinustaeda*, *Pinusvirginiana*	North America
Tortricidae	?*Rhyacioniasonia* Miller, 1967	*Pinusbanksiana*	North America
Tortricidae	?*Rhyacioniasubcervinana* Walsingham, 1879	*Pinusjeffreyi*, *Pinusponderosa*	North America
Tortricidae	?*Rhyacioniasubtropica* Miller, 1961	*Pinuselliottii*, *Pinuspalustris*, *Pinustaeda*	North America
Tortricidae	?*Rhyacioniazozana* Kearfott, 1907	*Pinuscontorta*, *Pinusjeffreyi*, *Pinusponderosa*	North America
Tortricidae	*Spilonotalaricana* Heinemann, 1863	*Larixaricina*, *Larixdecidua*, *Larixkaempferi*, *Piceasitchensis*	North America*, Europe
Tortricidae	*Tanivaalbolineana* Kearfott, 1907	*Piceaabies*, *Piceaengelmannii*, *Piceaglauca*, *Piceamariana*, *Piceapungens*, *Picearubens*, *Piceasitchensis*	North America
Yponomeutidae	*Cedestisgysseleniella* Zeller, 1830	*Pinuscontorta*, *Pinusmugo*, *Pinusnigra*, *Pinusnigrasubsp.Laricio*, *Pinussylvestris*	Europe
Yponomeutidae	*Cedestissubfasciella* Stephens, 1834	*Pinusmugo*, *Pinusnigra*, *Pinussylvestris*, *Pinusuncinata*	Europe
Yponomeutidae	*Ocnerostomafriesei* Svensson, 1966	*Pinusmugo*, *Pinussylvestris*	Europe
Yponomeutidae	*Ocnerostomapiniariella* Zeller, 1847	*Pinuscembra*, Pinuscontortavar.latifolia, *Pinusmonticola*, *Pinusmugo*, *Pinusresinosa*, *Pinusstrobus*, *Pinussylvestris*	North America*, Europe
Yponomeutidae	*Ocnerostoma* sp.	*Pinusresinosa*	North America
Yponomeutidae	*Ocnerostomastrobivorum* Freeman, 1960	*Pinusstrobus*	North America
Yponomeutidae	*Zelleriahaimbachi* Busck, 1915	*Pinusarizonica*, *Pinusattenuata*, *Pinusbanksiana*, *Pinuscontorta*, *Pinuscoulteri*, *Pinusechinata*, *Pinusengelmannii*, *Pinusjeffreyi*, *Pinusmuricata*, *Pinusponderosa*, *Pinusradiata*, *Pinusresinosa*, *Pinussylvestris*, *Pinustaeda*	North America
Diptera			
Agromyzidae	*Liriomyzaschmidti* Aldrich, 1929	?*Zamiaintegrifolia*	North America
Coleoptera			
Curculionidae	*Brachonyxpineti* Paykull, 1792	*Pinussylvestris*, *Pinusuncinata*	Europe

Note: Detailed information such as insect taxonomy, host taxonomy and local distribution are provided in Suppl. materials 1, 2. An asterisk marking the name of a continent (e.g. Europe*) indicates that the corresponding herbivore species is introduced in that region. A question mark "?" before an insect name indicates that this species has not been confirmed as a leafminer and a question mark "?" before a plant name indicates that its status as a host of the corresponding insect species requires confirmation.

**Table 2.** Number of gymnosperm-feeding leafminer species and number of host plant species used by these insects, in different leafminer families in North America (NA) and Europe (EU).

**Leafminer order**	**Leafminer family**	**Number of leafminer species in each leafminer family**	**Number of host species in each leafminer family**	**Leafminer genus**	**Number of leafminer species in each leafminer genus**	**Number of host species in each leafminer genus**
Lepidoptera	Tortricidae	53 (NA: 43, EU: 12)	64 (NA: 58, EU: 13)	*Rhyacionia*	19 (NA: 19, EU: 0)	21 (NA: 21, EU: 0)
				*Epinotia*	13 (NA: 8, EU: 6)	19 (NA: 15, EU: 6)
				*Choristoneura*	9 (NA: 9, EU: 0)	22 (NA: 22, EU: 0)
				*Argyrotaenia*	3 (NA: 3, EU: 0)	14 (NA: 14, EU: 0)
				*Archips*	2 (NA: 1, EU: 1)	20 (NA: 15, EU: 6)
				*Taniva*	1 (NA: 1, EU: 0)	7 (NA: 7, EU: 0)
				*Spilonota*	1 (NA: 1, EU: 1)	4 (NA: 2, EU: 3)
				*Pseudohermenias*	1 (NA: 0, EU: 1)	2 (NA: 0, EU: 2)
				*Dichelia*	1 (NA: 0, EU: 1)	2 (NA: 0, EU: 2)
				*Cymolomia*	1 (NA: 0, EU: 1)	2 (NA: 0, EU: 2)
				*Clavigesta*	1 (NA: 0, EU: 1)	3 (NA: 0, EU: 3)
				*Aethes*	1 (NA: 1, EU: 0)	1 (NA: 1, EU: 0)
	Gelechiidae	42 (NA: 41, EU: 4)	50 (NA: 45, EU: 10)	*Coleotechnites*	33 (NA: 33, EU: 1)	31 (NA: 30, EU: 2)
				*Exoteleia*	6 (NA: 6, EU: 1)	20 (NA: 18, EU: 4)
				*Chionodes*	2 (NA: 1, EU: 1)	7 (NA: 3, EU: 4)
				*Dichomeris*	1 (NA: 1, EU: 1)	2 (NA: 1, EU: 1)
	Argyresthiidae	20 (NA: 14, EU: 8)	28 (NA: 13, EU: 22)	*Argyresthia*	20 (NA: 14, EU: 8)	28 (NA: 13, EU: 22)
	Yponomeutidae	7 (NA: 4, EU: 4)	22 (NA: 17, EU: 7)
				*Ocnerostoma*	4 (NA: 3, EU: 2)	7 (NA: 4, EU: 3)
				*Cedestis*	2 (NA: 0, EU: 2)	6 (NA: 0, EU: 6)
				*Zelleria*	1 (NA: 1, EU: 0)	14 (NA: 14, EU: 0)
	Batrachedridae	2 (NA: 1, EU: 2)	7 (NA: 4, EU: 4)	*Batrachedra*	2 (NA: 1, EU: 2)	7 (NA: 4, EU: 4)
	Pyralidae	2 (NA: 2, EU: 0)	22 (NA: 22, EU: 0)	*Dioryctria*	1 (NA: 1, EU: 0)	11 (NA: 11, EU: 0)
				*Pococera*	1 (NA: 1, EU: 0)	11 (NA: 11, EU: 0)
	Coleophoridae	1 (NA: 1, EU: 1)	7 (NA: 6, EU: 7)	*Coleophora*	1 (NA: 1, EU: 1)	7 (NA: 6, EU: 7)
	Noctuidae	1 (NA: 1, EU: 0)	13 (NA: 13, EU: 0)	*Feralia*	1 (NA: 1, EU: 0)	13 (NA: 13, EU: 0)
	Blastobasidae	1 (NA: 0, EU: 1)	2 (NA: 0, EU: 2)	*Blastobasis*	1 (NA: 0, EU: 1)	2 (NA: 0, EU: 2)
	Adelidae	1 (NA: 0, EU: 1)	1 (NA: 0, EU: 1)	*Nemophora*	1 (NA: 0, EU: 1)	1 (NA: 0, EU: 1)
	Bucculatricidae	1 (NA: 1, EU: 0)	1 (NA: 1, EU: 0)	*Bucculatrix*	1 (NA: 1, EU: 0)	1 (NA: 1, EU: 0)
Diptera	Agromyzidae	1 (NA: 1, EU: 0)	1 (NA: 1, EU: 0)	*Liriomyza*	1 (NA: 1, EU: 0)	1 (NA: 1, EU: 0)
Coleoptera	Curculionidae	1 (NA: 0, EU: 1)	2 (NA: 0, EU: 2)	*Brachonyx*	1 (NA: 0, EU: 1)	2 (NA: 0, EU: 2)

**Table 3.** Number of host species and associated leafminer species in different gymnosperm plant families in North America (NA) and Europe (EU).

**Gymnosperm family**	**Number of host species in each gymnosperm family**	**Number of leafminers in each gymnosperm family**	**Gymnosperm genus**	**Number of host species in each gymnosperm genus**	**Number of leafminers in each gymnosperm genus**
Pinaceae	74 (NA: 65, EU: 29)	102 (NA: 83, EU: 26)	*Pinus*	38 (NA: 35, EU: 8)	67 (NA: 59, EU: 10)
			*Abies*	12 (NA: 7, EU:9)	31 (NA: 18, EU:13)
			*Picea*	10 (NA: 9, EU: 5)	28 (NA: 18, EU: 13)
			*Larix*	8 (NA: 8, EU: 6)	10 (NA: 8, EU: 4)
			*Tsuga*	4 (NA: 4, EU: 0)	9 (NA: 9, EU: 0)
			*Pseudotsuga*	2 (NA: 2, EU: 1)	7 (NA: 6, EU: 1)
Cupressaceae	26 (NA: 18, EU: 16)	32 (NA: 25, EU: 10)	*Juniperus*	14 (NA: 9, EU: 9)	22 (NA: 15, EU: 9)
			*Cupressus*	3 (NA: 1, EU: 2)	6 (NA: 4, EU: 3)
			*Thuja*	3 (NA: 2, EU: 3)	7 (NA: 5, EU: 4)
			*Chamaecyparis*	2 (NA: 2, EU: 2)	5 (NA: 2, EU: 4)
			*Calocedrus*	1 (NA: 1, EU: 0)	2 (NA: 2, EU: 0)
			*Sequoia*	1 (NA: 1, EU: 0)	1 (NA: 1, EU: 0)
			*Taxodium*	1 (NA: 1, EU: 0)	1 (NA: 1, EU: 0)
			*Platycladus*	1 (NA: 1, EU: 0)	1 (NA: 1, EU: 0)
Taxaceae	1 (NA: 0, EU: 1)	1 (NA: 0, EU: 1)	*Taxus*	1 (NA: 0, EU: 1)	1 (NA: 0, EU: 1)
Zamiaceae	1 (NA: 1, EU: 0)	1 (NA: 1, EU: 0)	*Zamia*	1 (NA: 1, EU:0)	1 (NA: 1, EU: 0)
