# Supplementary material for: A checklist of gymnosperm-feeding leafminers (Arthopoda, Insecta) in North America and Europe
Source: Biodivers Data J. 2022 Sep 28;10:e91313. doi: 10.3897/BDJ.10.e91313 (PMC9848575; doi:10.3897/BDJ.10.e91313)
Supplement: Supplementary material 3 — Insect families of leafminers on ferns, gymnosperms, angiosperms in North America and Europe [file bdj-10-e91313-s003.docx]

| **Host plant groups** | **Ferns** | **Gymnosperms** | **Angiosperms** |
| --- | --- | --- | --- |
| **Leafmining insect families** | **Coleoptera**: Curculionidae  **Diptera**: Agromyzidae, Anthomyiidae, Chironomidae, Pallopteridae  **Hymenoptera**: Tenthredinidae  **Lepidoptera**: Cosmopterigidae, Crambidae, Noctuidae, Psychidae, Tineidae | **Coleoptera**: Curculionidae  **Diptera**: Agromyzidae  **Lepidoptera**: Adelidae, Argyresthiidae, Batrachedridae, Blastobasidae, Bucculatricidae, Coleophoridae, Gelechiidae, Noctuidae, Pyralidae, Yponomeutidae, Tortricidae | **Coleoptera**: Attelabidae, Brachyceridae, Buprestidae, Chrysomelidae, Curculionidae, Megalopodidae, Nitidulidae, Rhynchitidae  **Diptera**: Agromyzidae, Anthomyiidae, Chironomidae, Dolichopodidae, Drosophilidae, Ephydridae, Phoridae, Scathophagidae, Sciaridae, Tephritidae  **Hymenoptera**: Argidae, Tenthredinidae  **Lepidoptera**: Acanthopteroctetidae, Adelidae, Autostichidae, Batrachedridae, Bedelliidae, Bucculatricidae, Choreutidae, Coleophoridae, Cosmopterigidae, Cossidae, Crambidae, Depressariidae, Douglasiidae?, Elachistidae, Epermeniidae, Eriocraniidae, Gelechiidae, Geometridae, Glyphipterigidae, Gracillariidae, Heliodinidae, Heliozelidae, Hesperiidae, Incurvariidae, Lycaenidae, Lyonetiidae, Momphidae, Nepticulidae, Noctuidae, Nolidae, Ochsenheimeriidae, Plutellidae, Praydidae, Prodoxidae, Psychidae, Pterolonchidae, Pterophoridae, Scythrididae, Tischeriidae, Tortricidae, Yponomeutidae, Ypsolophidae, Zygaenidae |

**Suppl. material 3. Insect families of leafminers on ferns, gymnosperms, angiosperms in North America and Europe**

? No leafminer species has been confirmed in Douglasiidae

**References：**

Yang J, Wang X, Duffy KJ, Dai X (2021) A preliminary world checklist of fern-mining insects. Biodiversity Data Journal 9: e62839. https://doi.org/10.3897/BDJ.9.e62839

Eiseman C (2022) Leafminers of North America. Second Edition. Charley Eiseman URL: http://charleyeiseman.com/leafminers/

Powell JA (1980) Evolution of larval food preferences in Microlepidoptera. Annual Review of Entomology 25 (1): 133‑159. https://doi.org/10.1146/annurev.en.25.010180.001025

Kristensen NP, Schmidt-Rhaesa A (1998) Handbook of Zoology. Lepidoptera, Moths and Butterflies.. Volume 1: Evolution, Systematics, and Biogeography. Berlin: Walter de Gruyter.

Ellis W (2022) Plant Parasites of Europe: leafminers, galls and fungi. URL: https://bladmineerders.nl/
